# KnobSlider: Design of a Shape-Changing Parameter Control UI and Study of User Preferences on Its Speed and Tangibility

**DOI:** 10.3389/frobt.2019.00079

**Published:** 2019-08-29

**Authors:** Hyunyoung Kim, Céline Coutrix, Anne Roudaut

**Affiliations:** ^1^LIG, Université Grenoble Alpes, CNRS, Grenoble, France; ^2^Department of Computer Science, University of Bristol, Bristol, United Kingdom

**Keywords:** shape-changing interfaces, parameter control interfaces, knob, dial, slider, design exploration, contextual inquiry, preference study

## Abstract

Professionals such as sound engineers or aircraft pilots heavily use physical knobs and sliders on their interfaces. The interfaces have advantages over touchscreen interfaces, especially when the users need to quickly and eyes-freely respond to changing situations such as when musicians are improvising, or there is smoke in a cockpit. However, unlike touchscreen interfaces, the physical interfaces are often bulky and crowded and lack of adaptability to user preferences or small spaces. To have advantages from both physical and touchscreen control interfaces, we explore design space of control interfaces and suggest design guidelines in the following steps. We first conduct a formative study with eight professionals who use knobs and sliders. Based on their feedback, we propose design requirements for future parameter control interfaces. We then introduce the design of the KnobSlider, a shape-changing device that combines the advantages of a physical knob and a slider in a time- and space-multiplexing way. To increase users' acceptance on shape-changing control interfaces, we investigate subjective preference on speed of shape-changes by using pairwise comparison with different maximum speeds. We also investigate how tangibility—showing KnobSlider on a video or showing it in the physical world—affects users preference and suggest speed design guidelines for future studies.

## Introduction

Many professionals (e.g., sound and light engineers, graphic designers, camera operators, and pilots) use physical controls to interact with a large number of parameters. The interfaces have evolved little in the past 30 years, and these still use physical controls despite touchscreen technology being widely used. In fact, physical interfaces are ideal for such professions as they provide haptic feedback and thus eyes free manipulation. Each type of controls has different interactive advantages: the most prevalent are knobs for fine adjustment and sliders for absolute positioning. Knobs, or dials (Michelitsch et al., 2004), are buttons controlled via rotation. Sliders are linear control elements consisting of rails and cursors. There is a variety of controllers, varying in angular or linear range, size, shape, torque or friction, with or without detents, and implemented via diverse technologies (of various resolution). Their interfaces allow users to simultaneously access a large number of parameters.

As a consequence of exploiting the advantages of physical controls, such interfaces are inevitably bulky and crowded. For instance, one of the sound systems we observed in this work offers more than 400 parameters through 3 banks of 112 controls−28 sliders with 4 layers each—and 65 knobs (**Figure 2**, P5–6). Such large interfaces are thus not portable, which can hinder users in many ways: e.g., sounds engineers cannot move around the stage to test sounds while adjusting parameters.

Furthermore, they are also cognitively demanding: for example, users must remember and reach the position of each parameter control. Touchscreen GUIs, which offer more flexibility by providing consecutively different interfaces on a surface, could solve some of these issues. However, they lack haptic feedback and hinder eyes-free interaction.

As a result, there is a tradeoff between the space occupied by input devices (e.g., sliders and knobs) and the number and types of controls they offer. Current solutions such as remappable banks or GUIs force users to choose either sticking to one type of device or losing physicality. In this paper, we offer *KnobSlider*, which provides both a physical knob and a slider through shape changing. It decreases the interface size and gains portability without losing the different types of controls or their physicality. We believe this is a strong advantage which was additionally suggested by our user population. Our bottom-up research procedure described below has the following contributions:

Through a formative study, we gain an **understanding of professional users' needs regarding parameter control**. We conducted contextual inquiries to learn about use of physical and touch screen controllers.

We derive **design requirements for a flexible physical interface element** based on the formative study. Users need fast, precise, eyes-free, and mobile interaction with a large number of parameters. They also need retro-compatibility with current interaction.

**Figure 1:**
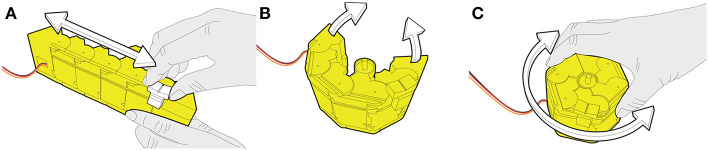
*KnobSlider* is a shape-changing device that changes between a rotational knob and a linear slider to accommodate users' needs. In the situation depicted **(A)** a sound engineer uses it as a slider to coarsely control a sound volume. She/he then presses the central button to change the device into **(B)** a low control-display (CD) gain knob, and **(C)** she/he can use it for fine adjustment.

We present **KnobSlider, a shape-changing physical control** that shifts between a knob and a slider (Figure 1). It combines the advantages of both types of controls while increasing the flexibility of the interface.

We report a **quantitative study on shape-change speed preference**. Results reveal that users prefer faster speed on videos than with physical device. Also, users least prefer the fastest speed, but once the shape-changes has started, they prefer the shape-changes finish quick. It suggests that future studies should use physical devices rather than videos, and once giving a sign to users for shape-changes, the device can have relatively fast shape-changes.

## Related Work

Our work aims to provide flexible physical interaction for the control of continuous parameters, through knobs and sliders. We survey previous works that investigated flexible and physical continuous input devices.

### Flexibility of a Single Device

An early height-changing knob was proposed by Hemmert et al. (2008). It was dedicated to cell phone notifications such as missed calls. Button+ (Kim et al., 2008) also changed the knob height to give distinctive control access to different users or to change the level of control difficulty for games. Haptic Chameleon (Michelitsch et al., 2004) was a shape-changing knob that changed its function according to its deformed shape to control videos. InGen (Badshah et al., 2011) was a passive knob with dynamic detent, stiffness, and abrupt stops to help users scroll a list. More recently, ExpanDial explored grasps of height- and diameter-changing dial (Kim et al., 2019), and DynaKnob (van Oosterhout et al., 2019) could change between four different knob shapes and dynamic force feedback.

Many studies have explored the flexibility of sliders focusing on dynamic haptic/force feedbacks. Some motorized slider cursors have been proposed for physics education (Shahrokni et al., 2006), haptic cues of sound amplitude (Shahrokni et al., 2006; Tanaka and Parkinson, 2016), and creating music loop (Gabriel et al., 2008). Vázquez et al. (2015) changed the haptic feedback of knobs and sliders by changing pressure in air chambers around the knob/slider axes. Zoomable TUIs explored physically zoomable sliders to balance between device footprint and pointing performance (Coutrix and Masclet, 2015). In addition to knobs and sliders, a volume-changing mouse was designed to allow zooming and scrolling through the control of the pressure (Kim et al., 2008).

### Space-Multiplexing

One approach to enable flexibility is to provide multiple devices in different locations simultaneously. There have been two approaches taken in spatially arranging controls. First, next to each other, like on sound mixing boards. Here, users manipulate sets of physical controls, including sliding and rotating joints (Blackwell and Edge, 2009). Their drawback is their footprint when space is a critical resource. Second, on top of each other, like in Zebra Widgets (Chan et al., 2012) and a rotary knob control for microwave oven (Djajadiningrat et al., 2007). They required less surface than the first arrangement. However, Zebra Widgets do not allow stacking a dial on a slider or vice versa. With the microwave control, moving one device might cause unwanted movement of the other device.

### Time-Multiplexing

Another approach is to have knobs and sliders in a sequence at the same location. With Paddle (Ramakers et al., 2014), users could make a flat surface for swipe (linear input), then deform it to a ring for rotational input. inFORM and Emergeables (Follmer et al., 2013; Robinson et al., 2016) could provide a slider and a knob at the very same location through the vertically actuated pins or circular “sensels.” With ForceForm (Tsimeris, 2013), users molded a placeholder to make a slider or knob on a touch surface. ChainFORM (Nakagaki et al., 2016) provided a linear or a round shape with actuation and touch sensing. However, in these implementations, the manipulation of the sliders was very different from that of the physical sliders, either lacking a physical cursor (Follmer et al., 2013; Tsimeris, 2013; Ramakers et al., 2014; Nakagaki et al., 2016) or continuity (Robinson et al., 2016).

### Both Space- and Time-Multiplexing

Some research approaches allowed both spatial and temporal multiplexing. With inFORM, Emergeables, and ForceForm (Follmer et al., 2013; Tsimeris, 2013; Robinson et al., 2016), it was also possible to provide several sliders and knobs sequentially in one place and simultaneously in different places through different implementations. For example, inFORM used an array of pins that can emerge to render sliders or knobs on the surface. However, the interactions with the interface are not same to the ones with knobs and sliders that professional users use. Instead of moving a slider cursor, the interface allows users to move their fingers on an emerged slider surface. Instead of rotating a knob, the interface allows users to move a token around the curved surface. Our approach also incorporates a combination of spatial and temporal multiplexing. In contrast to using widgets on touch surfaces (Weiss et al., 2009; Jansen et al., 2012), we want to avoid the need to place widgets on surfaces. In contrast to using discrete control points on a slider (Follmer et al., 2013; Tsimeris, 2013; Robinson et al., 2016), we allow for both continuous and physical manipulation of the cursor.

### Balancing Between Space- and Time-Multiplexing

Space- and time-multiplexing provide different advantages. Space-multiplexing allows spatial arrangements (Fitzmaurice and Buxton, 1997), persistence of attachment between devices and parameters (Fitzmaurice and Buxton, 1997; Badshah et al., 2011), exploiting spatial memory (Shaer and Hornecker, 2010; Scarr et al., 2013), simultaneous control of several parameters (Fitzmaurice and Buxton, 1997; Badshah et al., 2011), and specialized physical form factors (Fitzmaurice and Buxton, 1997). Time-multiplexing lowers hardware and maintenance costs (Fitzmaurice and Buxton, 1997) and avoids physical clutter (Shaer and Hornecker, 2010). Two extremes of these different multiplexing approached are “hundreds of potentiometers” and a “single mouse” (Badshah et al., 2011). Fitzmaurice *et al*. (Badshah et al., 2011) and Beaudouin-Lafon (Fitzmaurice and Buxton, 1997) say that the challenge lies in finding the optimal balance between the two types of multiplexing. We aim to fill in this gap and combine both multiplexing.

### Perceptional Studies on Shape-Changing Interfaces

Recently, studies on perceptional aspect of shape-changing interfaces are being conducted in HCI. Tiab and Hornbæk (2016) evaluated how movements of shape-changing buttons can provide affordances. They investigated how users perceive the status and interaction methods of 13 shape-changing buttons. The study shows that the participants did not perceive the movements of the buttons as the designers designed: they thought the buttons were showing warnings and did not think the buttons were “inviting” them to touch the buttons. Strohmeier et al. (2016) evaluate if shape-changing interfaces can convey emotions. They showed videos of a 3D modeled flexible phone to users and made them guess what kind of emotions the phone is showing through shape-change. The participants could recognize the emotions in the Circumplex Model by Russell (1980). However, the effect of the videos are unknown—we do not know if the result could would be better or worse if they used physical shape-changing devices.

## Formative Study

We first aimed to understand users' requirements in current practice of using control devices. To gather general requirements that encompass types of tasks, we decided to target various professions that require controlling interfaces, which include sound/light engineering, visual design, piloting, etc. To collect rich raw data regarding their workflows, we conducted contextual inquiry (Holtzblatt et al., 2004). It is a semi-structured interview method where instructors observe users performing tasks in their work environments and ask questions that are prepared and also spontaneously raised during the observation. Our observation and interviews of experts targeted specifically unresolved usability problems related to physicality and flexibility of devices for continuous parameters control—mostly knobs and sliders.

### Participants

We first identified the most widespread professions extensively using physical input control. According to the US Bureau of Labor Statistics^1^, in 2014 there were ~261,600 graphic designers, 119,200 pilots, 117,200 sound engineers, 20,060 camera operators, 11,930 exhibit designers (including light engineers). This approach allowed us to seek *importance* through a large population of users (e.g., graphic designers) and whose performance is critical to others (e.g., pilots); as well as to seek *generality* through diverse professions.

**Figure 2:**
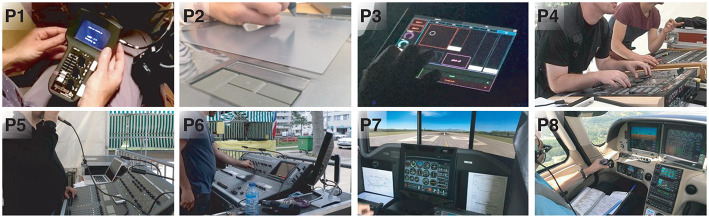
Participants using knobs and sliders in their professional activities: (P1) a camerawoman with 4 knobs and a slider on a custom made device; (P2) a graphic designer using a graphic tablet with a slider placeholder; (P3) a light artist using custom knobs and sliders on a tablet in a dark environment; (P4) a light engineer using physical knobs and sliders while observing a stage; (P5) a sound engineer communicating with musicians on the far stage while using sliders; (P6) a sound engineer controlling a knob while watching a screen; (P7) a pilot using flight simulator for his training; (P8) a pilot using physical controls in a flight.

Through our extended social network and calling/emailing local professionals, we recruited 8 participants (ages 25–63, 2 females, Figure 2) using control interfaces such as knobs and sliders in their professional activities: 1 movie operator (P1), 1 graphic designer (P2), 2 light engineers (P3, P4), 2 sound engineers (P5, P6), and 2 pilots (P7, P8). All participants were voluntary and consented to photo and video recording. The interfaces participants were using were mainly physical, although a few recently started using touchscreens.

### Procedure

All sessions took place in ecologically valid settings, and we observed all participants doing live activities. For example P1 was shooting a movie, P2 was drawing (P2), P3-6 were preparing a show, and P7-8 were piloting on a simulator or an aircraft (Figure 2). During the observation, we asked them to explain what was happening each time a sequence of actions was not clear. The activities were sometimes under a lot of pressure, for instance, a preparation of a show for the same night. When the participants required silence and concentration (P1, P3) and it was not possible to ask a question, we asked the action-related questions during the follow-up interviews. During the interview, we asked for situations where they needed to balance flexibility and tangibility. When possible, we conducted the interviews right after the activities. When it was not possible, the interviews were in the next morning. The observations and interviews took ~2 h.

### Data Collection and Analysis

We collected 8640 words of written notes, 141 drawings and photos (i.e., finger posture or devices used), and 2 h 34 m 26 s of video and audio recordings of particular sequences of actions or interview. When possible, we performed the analysis no later than 48 h after the interview. First, we described every sequence of observed actions. We used the collected notes, photo, and video recordings from our observations to help the description. We then used thematic analysis (Braun and Clarke, 2006) to analyze user needs regarding their controllers. We started with a research question: “what is needed for the users to perform their task?” A coder first labeled the observations with initial categories (codes) answering the question. An additional two coders joined to discuss, and agreed on them as well as identifying particular topics to regroup codes by themes. Our final scheme had six final main themes.

### Results

We identified six needs regarding the control of parameters. We illustrate them with examples of actions we observed or comments made by the participants.

Most knobs were potentiometers with no bounds or detents, of around 1 cm diameter with small lobes on their sides. We observed fewer varied knobs: e.g., with position mark (P1), concentric ones (P8), discrete arrow-shaped ones (P8) or large knob with a concave notch for rotation with a single finger (P4). In the following, we focus on the requirements that encompass knobs and sliders.

#### Interaction With a Large Number of Parameters

All participants interacted with a large number of parameters. The number varied depending on their professions. For instance, the fewest number of parameters were ten, which P1 (cinema operator) used to control the stereoscopic cameras' 3D position, 3D orientation, focal length, 1D focus distance, interaxial distance, and convergence. P2 (graphic designer) used as many of the ~60 Photoshop tools and their parameters (e.g., brush size, tip, roundness, hardness, etc.). During the show, P3 adjusted around 50 parameters in total. P4-8 (sound engineers, light engineers, and pilots) had more than 100 parameters to deal with.

#### Fast Interaction

##### Fast access to parameters

In many situations, the users needed to quickly acquire the devices. For example, to quickly access the parameters of a fan and a fog machine during the show, P3 (light artist) chose to permanently display two dedicated knobs on the left-hand side of her/his interface (Figure 2). P2 explained that her desk is always tidy: the participant needed a clear space to go from one device to another without losing time. P5 told that she/he never used the sliders that are too far away, and preferred pressing a button to quickly switch the parameters associated with the sliders that are close to her/him.

##### Fast manipulation of parameters

For instance, P7 related that the throttle was used by default for quick, coarse adjustments. P1, P3-P6 worked “live,” i.e., during the shooting or the show, so they must manipulate the parameters promptly. When working for music concerts, they needed to be very reactive as musicians never play the same way. The need for fast manipulation required some participants (P3-P6) to use several fingers on sliders or two hands on different devices. P2 explained that she/he started using the computer to work faster: P2's work requires several back and forth exchanges with the client who asks for modifications. P2 used the computer to do quick corrections (undo) that physical brushes and pens could not.

##### Fast observations of parameters

For example, P1 was manipulating interaxial distance between stereoscopic cameras and the bounds of the slider were clearly showing her the physical constraints of cameras. P1 also used red tape to mark a particular value of the interaxial distance during the shooting (see Figure 2 P1, the left side of the slider). P5 and P6 sometimes quickly glanced at their interfaces to observe a parameter value during the show. During manipulation, the knobs of the cockpits (P7-P8) provided haptic feedback through haptic detents. For quick observations of parameters, P7-P8 looked or touched the corresponding devices. P7 explained that in emergency situations, quick observation of parameters is critical. Overall, sliders were preferred for rapid observation of parameters.

#### Precise Interaction

For precise interaction, most participants used large sliders (~8–10 cm), with very smooth friction to allow tiny movements. The only small slider we observed was a tactile one with a placeholder (Figure 2, P2), to zoom in on the screen. When operating a slider, P1 placed her hand carefully to avoid mistakenly moving the dials next to it (Figure 2).

Knobs, even when small, also offered high precision as most of them were multiturn. Using the knob for precise control was done by P2 who used her tactile dial with a placeholder to scroll webpages. We observed P4 (light engineer) using a knob before the show to very precisely set a projector angle (Figure 2, P4). For this, P4 performed many rotations on a knob with low control-display (CD) gain. Similarly, P7 and P8 (pilots) used knobs to accurately input decimal values of radio frequencies. P7 related that the extremity of her/his aircraft's throttle could be turned for precise adjustments. P6 needed to adjust the curve of sound level (dB) for each frequency (Hz) of each instrument and microphone on stage. The participant preferred using the physical knobs for this rather than the touchscreen. P1 (movie operator) used a knob to adjust, at pixel precision, the horizontal shift between stereoscopic images, by performing multiple rotations. Overall, knobs were preferred for precise interaction.

#### Eyes-Free Interaction

P1 needed eyes-free interaction because her screens were not collocated with her knobs and sliders. Similarly, P2 focused on her canvas on the screen and looked away to modify her tool, thus lost time finding her object on the canvas again. P3-P6 watched the stage while interacting (Figure 2). P5 explained that she/he preferred physical controllers, and never used the touchscreen of the console, which our observations corroborated. P5 and P6 said that interacting with bounded parameters on multiturn—i.e. unbounded—knobs was not comfortable, as they have to look away from the stage to watch the LEDs around the knob. Bounded knobs were not available on the mixing console we observed, and participants said that they also seldom find them on other mixing consoles.

P3's problem with touchscreens was that she/he felt “blind” as there was no haptic feedback. We observed P3 missing an intended trajectory of a knob on the screen: the participant started to follow the knob on the tablet. While looking at the stage, P3 drifted from the knob, losing control of it. When the participant realized it, she/he looked down to reacquire the knob. We observed the participant trying to grasp two touchscreen sliders eyes-free: one with the index finger and the second with the middle finger, both unsuccessfully. P3 then looked down to re-grasp the sliders. The participant recouped the lack of tangibility with extra-large widgets, but it was not satisfactory for her/him: the participant lost space, and still lacked tangibility, which caused critical errors.

P7 and P8 (pilots) used push/pull handle for power and mixture (of air and fuel) and often placed their hand on the handles to know their status without looking (Figure 2, P8). P7 said, “If you put your hand on [the control device], you know in which mode you are.” They commented that physical devices were particularly useful when visibility in the cockpit was altered by smoke. P7 and P8 both explained that aircraft manufacturers were introducing touch screen interfaces. Both agreed that the idea was dangerous. Their comments strengthened the cockpit design requirements in previous work (del Castillo and Couture, 2016; Vinot et al., 2016).

#### Mobile Interaction

All users needed mobility. P1 used her cameras and control devices at different locations. P2 sometimes worked away from her desk, e.g., in a van during a vacation. P3 explained that moving around the stage was crucial for her/him: when not possible, she/he communicated with someone in front of the stage as a proxy. Unfortunately, this person might not understand what the participant wanted or did not have the same demand on the final quality. To avoid losing time or quality, P3 used a tablet, but sub-optimally moved back and forth between her/his desk and the stage. P4 went on the stage to better see the projectors, and then came back to the console. We observed P5 going on the stage to ease the communication with musicians. P5 said this was the only reason she/he used the tablet, as she/he did not like using the sliders/knobs on touch screens. To avoid using the tablet, the participant communicated with musicians via a microphone (Figure 2, P5), or even shouted or signed. Sometimes a third person was necessary to help communication with drummers who did not have a microphone. Thus, all solutions were suboptimal. Mobility was also necessary during the show: P6 walked around the venue to hear the sound from other locations. P6 then had to come back to the mixing desk to adjust the parameters. P7 and P8 (pilots) had a compact interface that fits in the cockpit.

#### Retro-Compatibility

The professional users needed to leverage their existing expertise with current interfaces. For example, P2 explicitly commented that she/he was not keen to change her/his interface to another, because her/his workflow was efficient and she/he was not ready to lose income for a short term. Similarly, P5 did not use her/his tablet as much she/he used her/his mixing console, as the tablet lacked retro-compatibility, such as tangibility. The only participant that explicitly showed no interest in retro-compatibility was P3. P3 built a new interface dedicated for each show and improved it with practice. The participant used the touchscreen interface and laptop, and tried to use new technologies such as Leap motion although she/he did not have time to set it up and use it at our observation. Yet, her/his touchscreen controllers were mimicking physical ones (knobs, sliders, buttons, etc.).

### Design Requirements for Control Interfaces

We derived the following six requirements directly from the themes of our formative study.

*R1. Interaction with a large number of parameters*. All participants needed to control lots of numbers of parameters through their workflows. The cameraman had the least (10). The sound and light engineers can deal with more than 100. Types of parameters were diverse: some were discrete (e.g., tool in Palette) or continuous (e.g., sound volume). Some were bounded (e.g., flaps' angle) or not (e.g., shift between cameras). Some were cyclic (e.g., projector's angle).

*R2. Fast interaction*. The participants needed to control parameters quickly. To do it, they needed quick access to, rapid manipulation of, and fast observation of parameters. Quick access to parameters can be supported by placing devices within users' reach. Rapid manipulation of parameters can be supported through smooth trajectories. Fast observation of parameter value can be carried by visual and/or haptic display, including min/max value or value of interest.

*R3. Precise interaction*. The participants also needed precise control of parameters. It can be supported through a large interaction area (multiturn knob or large slider) and little friction. Enough space between devices prevents errors. A stable grip on the device also allows its operation without slipping.

*R4. Eyes-free interaction*. The participants needed to observe how the controlled parameters affect the stage, video, graphic, sound, etc. while interacting with the interfaces. Eyes-free access to parameters can be supported by spatial stability of the device to leverage motor-spatial memory. Eyes-free manipulation of parameters can be supported through physical trajectory guide (e.g., slider's rail, knob' rotational axis) and haptic feedback. Eyes-free observation of parameters' value can be supported through a physical cursor or haptic feedback (detent).

*R5. Mobile interaction*. Some of the participants such as the light engineers and cinema operator needed to move around the workplace to check the audiences view and to support scenes where cameras move. Mobile interaction can be supported by small devices, such as the light engineer used his tablet to control some parameters in front of the stage.

*R6. Retro-compatibility*. The participants are professional, and new interfaces need to support them with current interaction: it is arduous for users to give up current UIs—even though new ones can be beneficial in the long term (Scarr et al., 2011). This can be supported by standard operations of standard devices and customizability.

Some of these requirements are incompatible (e.g., a slider should be large for precision and small for mobility). As a consequence, a good design needs to find a compromise in order to maximize users' satisfaction.

## KnobSlider Design

Our formative study shows that the users' needs for tangibility and flexibility are hardly addressed in current systems. To address this problem, we propose to design a device that is a self-transformable input device that can be changed to provide an interface on-demand from a knob to a slider and vice versa. This section presents our initial exploration for the design of such shape-changing device. In particular, our approach to find the best design candidate and to implement it consists in three steps described below.

We explore design space of existing control interfaces in terms of space- and time-multiplexing. We then specify where we target in the design space.

We create low-fidelity prototypes by using ideas inspired from deformable artifacts found in daily objects or on fabrication and DIY websites. We then analyze them using the design requirements from the formative study. The summary of the analysis is in Table 2.

We implement a working prototype KnobSlider, based on the analysis of the low-fidelity prototypes.

### Target Design Space

We focus on the physical interfaces that have some retro-compatibility (R6) with physical knobs and sliders (rotational/linear interaction). They have different spatial and temporal combinations as shown in Table 1.

**Table 1:** Design space and related work of control interfaces, in the perspective of time- and space-multiplexing.

	***No time-multiplexing:* Device(s) available all the time**	***Time-multiplexing:* Devices available in sequence**
***No space-multiplexing**: One* device available at the workspace	*A*. A single knob or slider (Michelitsch et al., 2004; Coutrix and Masclet, 2015; Vázquez et al., 2015; Suh et al., 2017)	*B*. A knob and a slider in sequence (Follmer et al., 2013; Tsimeris, 2013; Ramakers et al., 2014; Nakagaki et al., 2016; Robinson et al., 2016)
***Space-multiplexing**:* Multi-devices available at the workspace	*C*. Adjacent knobs and sliders and stacked knobs and sliders (Djajadiningrat et al., 2007; Blackwell and Edge, 2009; Chan et al., 2012) and current systems	*D*. Knobs and sliders anywhere, anytime: (Weiss et al., 2009; Jansen et al., 2012; Follmer et al., 2013; Tsimeris, 2013; Robinson et al., 2016) and current systems

**Table 2:** Summary of the low-fidelity prototype idea evaluation based on the design requirements.

**Design requirements**	**Low-fidelity prototype ideas**									
	**1**	**2**	**3**	**4**	**5**	**6**	**7**	**8**	**9**	**10**
R1. Interaction with a large number of parameters	•	•	•	•	•	•	•	•	•	•
R2. Fast interaction	•	•	•	•	•		•	•		•
R3. Precise interaction	•			•		•	•	•	•	
R4. Eyes-free interaction	•	•	•	•		•		•	•	•
R5. Mobile interaction		•	•	•	•				•	•
R6. Retro-compatibility	•	•	•	•	•	•	•	•	•	•
Total score	5	5	5	6	4	4	4	5	5	5

The current solutions largely cover the design space but lack some of the requirements:

*A*. A single knob would hinder the fast and eyes-free operation of a parameter (R2, R4). A small single slider would either hinder precision (R3) and a large single slider would take too much space (R5) on a surface.
*B*. A knob or slider sequentially morphing out of a surface currently lacks continuity (R1) or physical cursors (R4). Manually placing a knob or a slider on a surface is time-consuming (R2).
*C*. Adjacent knob and slider are space-consuming (R5). A knob on top of a slider (and vice-versa) would cause unwanted movement thus lack precision (R3).
*D*. Physical knobs and sliders anywhere, anytime are not fully supported yet. As in B, manually placing knobs and sliders on a surface is time-consuming (R2) and knobs and sliders morphing out currently lacks continuity (R1) or physical cursors (R4). Current systems partially support time-multiplexing through banks of sliders only.

We aim a novel solution improving the tradeoff between users' requirements: a device that takes the shape of either a knob or a slider in sequence, improving time-multiplexed solutions (B). Several of such devices combined will improve time- and space-multiplexed solutions (D).

### Low-Fidelity Prototype Exploration and Their Analysis

**Figure 3:**
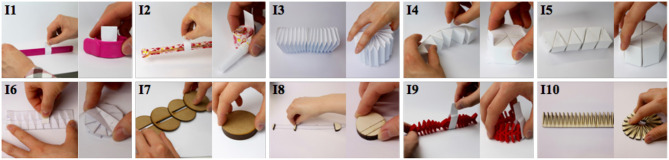
Two states of low-fidelity prototypes: slider (left) and knob (right). (1) slap bracelet, (2) party whistle, (3) accordion, (4) roly-poly, (5) twisty toy, (6) origami, (7) stackable disk, (8) zip-line, (9) fish bone, (10) dukta pattern.

To provide a solution to the design requirements from the formative study, we explore initial designs with time- and space-multiplexing features. We created low-fidelity prototypes using deformation ideas inspired from deformable artifacts found in daily objects or on fabrication and DIY websites^2^ (Figure 3). We present each prototype in the following two aspects.

1. *Principle*: describes the low-fidelity prototype and how it can make a linear shape to be used as a slider and make a round shape to be used as a knob. We also describe the key mechanical or materialistic features that would affect the shape-change implementation and user interaction.
2. *Design*: illustrates the envisioned implementations that suits the low-fidelity prototypes. To choose the implementation method, we first considered three actuation method that are widely used for shape-changing interfaces—rotational motor (Nakagaki et al., 2016), pneumatic actuation (Blackwell and Edge, 2009; Follmer et al., 2012; Yao et al., 2013), shape-memory alloy (Roudaut et al., 2013)—and then chose the best actuation method for each design.
3. *Analysis*: reports how the low-fidelity prototypes satisfy or do not satisfy the design requirements based on their current designs and the envisioned implementations. For *R1. Interaction with a large number of parameters*, we count how many parameters the prototype can support without banks. For *R2. Fast interaction*, we consider how fast the shape-change between the knob and slider states would be. It is dependent on the actuation methods. For *R3. Precise interaction*, we consider the both knob and slider states of the device. First, it is desired to have multi-turn knob to allow low CD gain. We evaluate if the designs can allow multiturn of the knob. Second, it is desired to have a long slider. We evaluate the ratio between the slider length and the knob circumference. We decide the minimum desired ratio as 1:1 and the bigger the ratio the better the system can support precise interaction. When evaluating if the prototype can support *R4. Eyes-free interaction*, we look at the slider cursors. By having a physical slider cursor, users can continue controlling the slider without looking at it once they grab it. *R5. Mobile interaction* refers to the fact if the device can be used in mobile context, e.g., while users are moving around a stage. Lastly *for R5. Retro-compatibility*, we see if users can interact with the prototypes in the same way they interact with currently available commercial interfaces.

#### Idea 1: Slap Bracelet

*Principle*: The idea is to use a bi-stable strip, such as slap bracelets, bicycle ankle bands, or tape measurers. Such objects consist in a bi-stable material that can be in two stable shapes: rolled or straight. When rolled it can be used as a knob and when straight it can be used as a slider.

**Figure 4:**
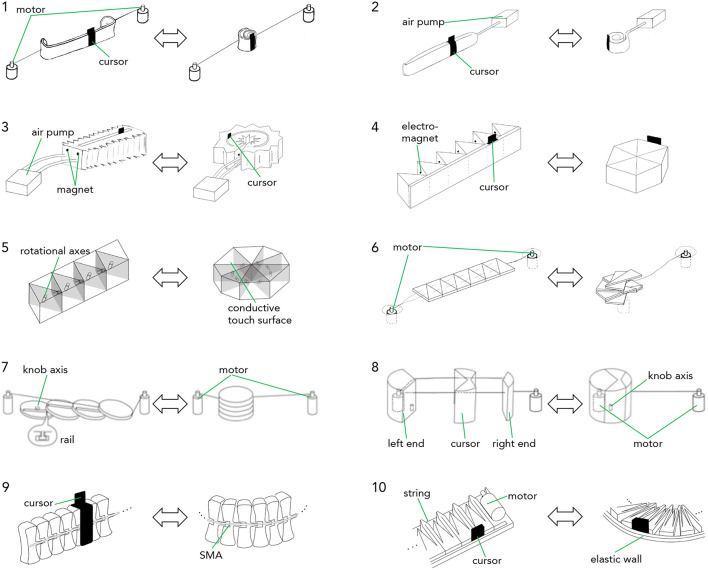
Hypothetical actuation methods for the low-fidelity prototypes. The left shows slider state and the right shows knob state of each prototype. Idea 1 (slap bracelet) with motor actuation. The motors pull strings to make the slider (left), and loosen them to make the knob (right) Idea 2 (party whistle) with pneumatic actuation. The air pump inflates to make the slider (left), and deflates to make the knob (right). Idea 3 (accordion) with pneumatic actuation. The air pump puts the same amount of air into two chambers to make the slider (left). It inflates the outer and deflates the inner one to make the knob (right). Idea 4 (roly-poly) with electromagnet actuation. The magnets pull neighbor prisms to transform to the knob (right). When they change polarity, it goes back to the slider (left). Idea 5 (twisty toy) with motors. Motors rotate each face to transform the slider (left) to the knob (right). Idea 6 (origami) with motor actuation. The left motor pulls strings to fold the object to the knob (right). The right motor pulls the strings back to make the slider (left). Idea 7 (stackable disks) with two motors. The right motor pulls the top-most disk to make the slider (left). The left motor pulls it back to make the knob (right). Idea 8 (zip-line) with motor and magnetic actuation. The motor pulls the strings to make the knob. An under-table plotter with magnetic head moves the right end to make the slider. Idea 9 (fish bone) with Shape Memory Alloy (SMA) actuation. When SMA is heated, the bone bends and make the knob (right). When the SMA is cooled, the bone goes back to the straight slider (left). Idea 10 (dukta pattern) with motor actuation. The motor pulls the string to form the knob (right), and loosen it to form the slider (left).

*Design* (Figure 4-1): The prototype could be actuated using two rotational motors. The motors are fixed on a surface and connected to each extremity of the strip via strings. When the motors rotate outward, they pull the strings and make the strip to be straight, except at its extremities. The slightly rolled extremities stabilize the slider on the surface and also enable the slider to go back to the knob state when the motors move inwards. We can place a slider thumb around the bracelet. When the motors move inwards, the strip rolls back by its bi-stable property.

When the motors are actuated in the opposite direction, the band comes back to a roll. By letting the string loose enough, it is also possible to perform rotational movements with the knob. The cursor is a piece of material that fits around the band and that slides along it. In a knob position, the cursor does not move anymore. Note that it would be possible to use only one motor because the band behaves like a spring and can come back to the roll if it is not maintained in tension. However, such solution would require the band to be fixed on a surface, which is not ideal for mobility.

*Analysis*: (R1) The design supports users to control two parameters with the knob and slider shapes. (R2) When the device changes its shape from slider to knob, the shape-change would be fast as the slap bracelet has the property of rolling fairly fast when it is bent a bit. The change from the slider to the knob would be fast as well as the motors can actuate it fast. (R3) The slap bracelet can have the slider with variable length, including the length longer than the knob circumference. (R4) The device has the cursor and hence allow eyes-free interaction during the slider status. (R5) The actuation motors are fixed on a surface, and it would be hard to make the interface mobile. (R6) The design allows the same interaction of rotating the knob and sliding the slider cursor from current practice.

#### Idea 2: Party Whistle

*Principle:* the idea is to use a party whistle mechanism, which is rolled in its initial shape (knob), but become linear when inflated (slider).

*Design* (Figure 4-2): the prototype could be actuated using pneumatic, i.e., using an air pump connected to the whistle in order to change from slider (inflated) to knob (deflated). A ringed cursor can surround the inflated part. The ringed part of the cursor should be deformable enough in order for the cursor to fit the whistle diameter even when inflated.

Analysis: (R1) The design allows controlling two parameters from the two states. (R2) The pneumatic actuation would allow fast shape-change from knob to slider and vice versa. (R3) The range of the rotational interaction would be limited by the air tube, as its length would be limited to keep the size compact and the tension of the air tube would rotate the knob unintentionally. The ratio between the slider axis and knob circumference can be more than 1 and allow precise control. (R4) The slider cursor would allow eyes-free control. (R5) The compact design and the pneumatic actuation would allow the device used in mobile scenarios (Follmer et al., 2012), although the noise from the air pump could decrease applicable usages (e.g., noise during shooting a movie). (R6) The rotational and linear interactions with the prototype is same to the ones with current control devices.

#### Idea 3: Accordion

*Principle:* the device consists of a rectangular prism whose structure use accordion origami folding. This structure allows the structure to be elongated and bent as shown in Figure 3. When elongated it create a slider but when bent, it is possible to create a circular knob.

*Design* (Figure 4-3): the deformation could be done using pneumatic actuation by using two adjacent chambers that are running along the elongation axis. By inflating both chambers simultaneously, it allows to elongate the device, however when inflating only one chamber while deflating the other (shrinking it), the device can transform into a circular knob. Magnets or electromagnets at each end of chambers can then complement the pneumatic actuation and lock the device in the form of a knob. Creating a groove in the middle of the device can allow placing a cursor whose supporting structure touches the bottom of the device.

*Analysis*: (R1) The design allows controlling two parameters with the linear and round shapes. (R2) The pneumatic actuation would allow fast shape-shifting. (R3) Similarly to the party whistle design, it would have the air tube around the knob circumference and it would be hard to implement multi-turn knob. However, the axis of the slider can have the same length with the knob circumference and allow relatively precise interaction at the slider status. (R4) The cursor on the slider would allow eyes-free interaction. (R5) Air pump can be small enough to support mobile interaction (Follmer et al., 2012). (R6) The interactions with the design stay linear and rotational, which is same with the control devices in practice.

#### Idea 4: Roly-Poly

*Principle:* a student design work (Fulda et al., 2014) inspired our design. The idea is to use six triangular prism modules connected to each other as shown on Figure 4. When folded the prisms form a hexagonal prism (knob) but when unfolded the prisms are aligned, thus creating a support for the slider cursor. It is for example possible to add an external wall (see Figure 4-4) on which the cursor can slide.

*Design* (Figure 4-4): we see two possible ways to implement this idea. The first one using motors, the second one using electromagnets. In the motor implementation, a motor could be placed inside the modules (except one) to perform the folding and unfolding. In the electromagnet implementation, a pair of electromagnets is placed inside each module. One electromagnet is used for attracting the prism together while the other one is used to repulse them. The material used to create the external wall could have a certain tension in order for the device to “snap” into the slider form.

*Analysis*: (R1) Each knob and slider status can support controlling one parameter. (R2) The motors in the device would allow fast shape-change between the two statuses. (R3) There is no air tube around the knob circumference, which allows boundless rotation. The length of the slider axis is similar to the knob circumference, which allow precise control on the slider without losing much space. (R4) The design also has a physical cursor, so that users can continue manipulating it without visual attention once they grab it. (R5) The motors are only attached to the device and not on the surface. It would users to take the device on-the-go and use it on any surfaces including their palm. (R6) Similarly to other designs, it allows rotational and linear controls.

#### Idea 5: Twisty Toy

**Figure 5:**
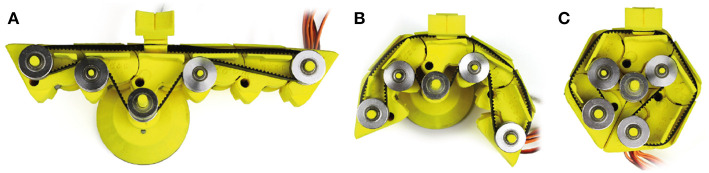
**(A–C)** KnobSlider working prototype without the top cover to expose the slider's timing belt and the slider mechanism. **(A)** In slider shape, the movement of timing belt is conveyed through the gears, **(B)** during transformation, the edges of blocks start to lock the bottom central gear, **(C)** the edges completely lock the bottom central gear, the rotation of the knob does not affect the timing belt.

*Principle:* the idea is to use a similar mechanism used in toys such as the ShengShou Magic Snake. The device is made of triangular prism modules that are connected to each other through a rotary joint (Figure 4-5). Contrarily to idea 4, the modules are connected by their faces thus allowing the device to deform between a hexagonal prism (knob) and an elongated rectangular prism (slider).

*Design* (Figure 4-5): one motor can be placed in each module (except the last one) and would perform the rotation between two parts of the structure. It would be complex to add a groove to place a physical slider but capacitive sensors covering the prisms could serve as input for sliding interaction.

*Analysis*: It has similar properties than the idea 4 roly-poly: (R1, R2) It can support controlling two parameters; (R5) the motors in the device would enable fast shape-changes and mobile interaction; and (R6) the rotational and linear interaction would be retro-compatible. (R3) The device would not have air tubes around it, so that users can have a multi-turn knob. However, the length of the slider would be the half of the knob circumference. (R4) Also, it cannot have a physical slider cursor as the faces of each element will change the directions during shape-changes.

#### Idea 6: Origami

**Figure 6:**
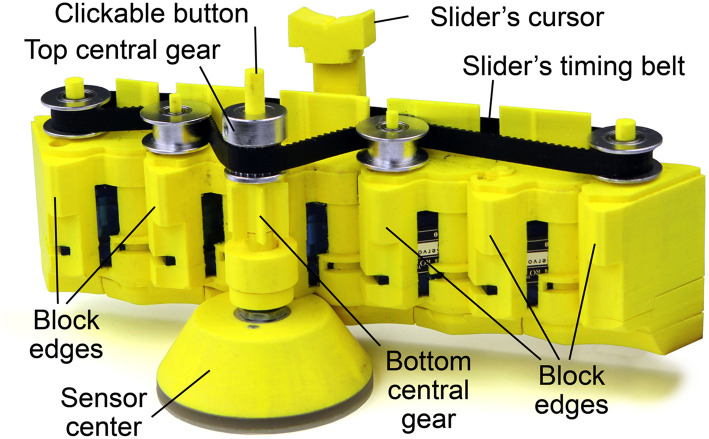
Elements of KnobSlider, here without the top cover to expose the slider's timing belt.

*Principle:* the idea is inspired by an origami pattern (Jackson, 2013) where a rectangular shape is folded in small triangles in zigzag manner (Figures 3–6). The slider shape is a flat rectangle and the knob is a folded polygon (knob). In order to approach the shape of a circle for the knob, many folds are needed.

*Design* (Figure 4-6): instead of using a piece of paper it would be more practical to laser cut a series of triangle shapes made of two layers and to attach them together using a layer of flexible plastic crushed in between the two layers. A cable or tread is then stitched along each piece. To fold the prototype to the knob state, one motor pulls both threads while the bottom triangle is maintained fixed. To unfold the prototype to the slider state, another motor drags the top triangle back to its initial position. A tangible cursor can slide along a central groove (Figures 3–6, left). This tangible cursor should be able to flatten before transforming to the knob state in order to allow the prototype to fold smoothly.

We did not consider SMAs to implement this idea. The reason is that it is really difficult to educate an SMA to fold at 180 degrees without industrial machineries (the material is too weak and break).

*Analysis*: (R1, R6) the round and linear shapes support controlling two parameters and retro-compatibility. (R2) The motors on the surface would allow fast shape-changes, but (R5) it would be hard to support mobile interaction. (R3) There would be tendons around the knob, but they can to elastic. If they are long but thin enough to be coiled around the motor axes, users can have a multi-turn knob. The minimum ratio between the slider length and the knob circumference is 0.5:1, but it can be easily increased and go over one when we add the triangle shapes. (R4) We embedded the physical cursor on the slider, and it would allow eyes-free interaction.

#### Idea 7: Stackable Disks

**Figure 7:**
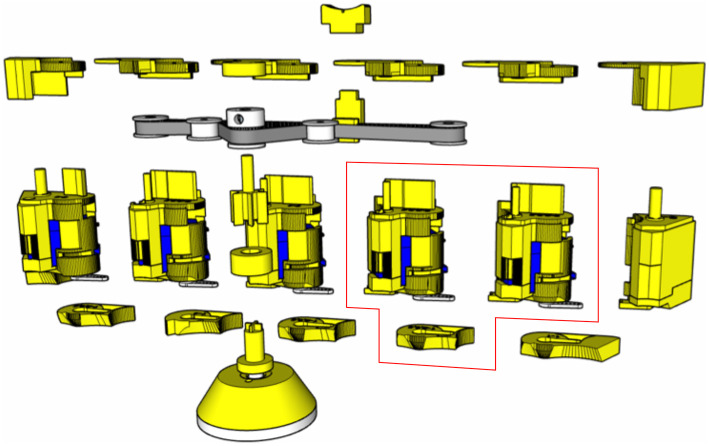
The assembly view of KnobSlider. The device consists of forty pieces of the printed case (yellow), five pulleys, a timing belt, a rotational sensor, five servo motors (blue).

*Principle:* the device consists of series of stackable disks. When the disks are stacked up they form a knob. Below each disk is a knob that slides into the groove of the lower disk (Figure 4-7). This mechanism allows to unstack the disks and to form a slider to a knob.

*Design* (Figure 4-7): the lowest disk is fixed on the support surface with a rotation axis to support interaction with the knob state of the device. In order to (un)stack the disks, two motors drag away(back) the top-most disk along the slider axis via strings. To operate the slider, the user slides her/his finger on the unstacked disks.

*Analysis*: (R1, R6) The stacked disks can support rotational control, and the unstacked disks can support linear control. Similar to Idea 6 Origami, (R2, R5) The motors on the surface would support fast shape-changes but not mobile interaction. (R3) It would be also possible to have a multi-turn knob thanks to elastic and then tendon. Also, the design can have the ratio of more than one, as it is easy to add more disks. (R4) However, it would be hard to embed a physical cursor for the slider state because it is not clear how the cursor can travel between separate disks.

#### Idea 8: Zip-Line

**Figure 8:**
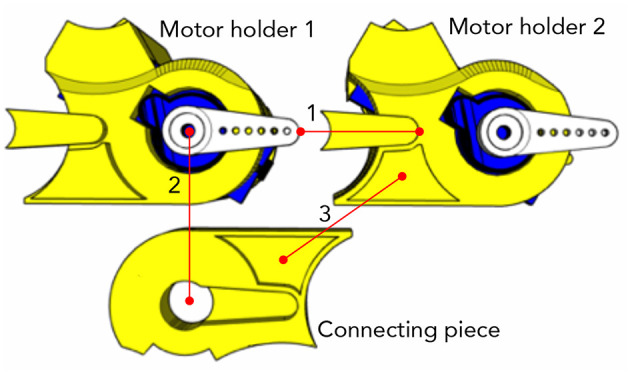
A closer look at the piece in the red box in Figure 7, to show how two motor holders are connected through the connecting piece. They are showing the bottoms of the motor holders and top of the connecting piece. The connecting piece is showing the top to show the grooves for connection. The dots on the red lines indicate which part should meet which part when assembled. (1) The prominent part of the motor holder 2 goes under the servo motor arm on the motor holder 1. (2) The groove on the connecting piece looking like the servo motor arm locks the prominent part and the servo motor arm. (3) The second groove on the connecting piece additionally holds the motor holder 2.

*Principle:* the idea is to use a knob that can breaks into three pieces attached with a double string. The middle pieces slide along the string thus creating the slider (Figure 4-8, left). The left end piece is large enough to include the rotational axis of the knob, which is fixed on the support surface.

*Design* (Figure 4-8): the prototype could be actuated using a single motor, which pull the right end of the device, the left end being anchored. A motor placed in the left end would serve to pull on the string in order to deform the device back into a knob. The structure can be maintained in slider position using magnets underneath the surface.

*Analysis*: (R1, R6) the combined pieces would work as a knob, and the separated pieces would work as a slider. (R2, R5) It has two motors on the surface, and it would have quick shape-changes but not be used in mobile context, same to Idea 7 Stackable disks. (R3) It can have a multi-turn knob if the motors keep the tendons loose during the knob status. It can also have a long slider axis as much as the surface allows. (R4) The middle piece acts as a slider cursor and provides eyes-free interaction.

#### Idea 9: Fish Bone

**Figure 9:**
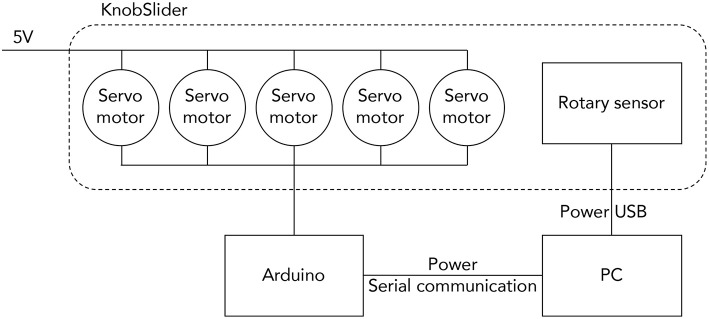
The system diagram of KnobSlider. Five servo motors are powered by an external source (5V). They are controlled by an Arduino, and the Arduino communicates with a PC. The rotary sensor in KnobSlider communicates with the PC separately.

*Principle:* a fish bone model^3^ inspired our design. Each bone is connected to the next one like in the chain of a bike (Figure 4-9). The resulting structure is highly flexible, which is a very interesting aspect because it would be used to create sliders that are non-linear like the virtual ones proposed in SketchSliders (Tsandilas et al., 2015).

*Design* (Figure 4-9): two SMAs could actuate the design, one on the inner circle surface to fold into the knob state; and the other on the outer circle surface to unfold into the slider state. This would require educating the SMA's to bend to a certain shape. As with idea 2 party whistle, the slider cursor can be wrapped around the structure and could move along it.

*Analysis*: (R1, R6) the roundly bended shape supports a rotational manipulation, and the straight shape support a linear manipulation. (R2, R5) The actuation of the SMAs would be slower than the motor or pneumatic actuations. However, the compactness of the SMAs would allow mobile interactions. (R3) There would be no air tube around the knob shape, and users would be able to turn the knob multiple times. The ratio between the slider and the knob is one, but it can be larger when the knob makes a spiral. (R4) The device can have a physical slider cursor and hence support eye-free interaction.

#### Idea 10: Dukta Pattern

**Figure 10:**
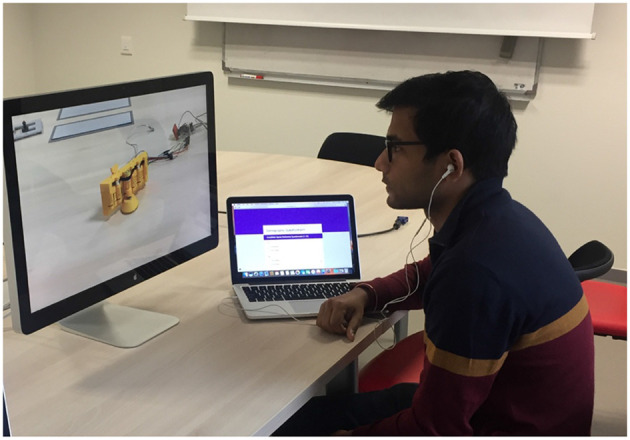
A participant in the *video* condition. She/he is looking at a video of KnobSlider changing its shape on a screen. The size of the device on the screen is controlled to have the same size in the *device* condition.

*Principle:* dukta or kerfing cut patterns allow creating flexible material although using rigid materials such as wood. Through the fine-tuning of the pattern's parameters, bending direction, and degree can be fine-tuned. For example, it is possible to use this patter to go from a rectangle shape (slider) to a disc (knob) as shown in Figure 4-10.

*Design* (Figure 4–10): a string could be stitched along one side of the slider (at the center). A motor placed at one end of the structure would pull the string in order to fold the structure into the knob state. A physical cursor could be placed between two dukta patterns.

*Analysis*: (R1, R6) the device enable users to have linear and rotational interactions. (R2, R5) The motor on the patterns would allow fast shape-changes and mobile interactions. (R3) Ideally there should be nothing around the pattern when it has a battery on it. It would allow multi-turn of the knob status. However, the knob would have longer circumference than the slider axis, as the outer edge of the pattern would expand when it is bended to a knob. (R4) The device can have a physical cursor when there is an elastic wall at the outer axis.

### Working Prototype Implementation

Through the evaluation of the low-fidelity prototypes, we converged on a particular design that better supports the requirements. The design has six triangular prism blocks connected to each other (Figures 5A–C). When folded (C) the prisms form a hexagonal prism (knob). When unfolded (A), the prisms are aligned, thus creating a connected surface. A cursor can move along the surface. The design is originally inspired by the Sensitive Rolypoly (Fulda et al., 2014), and the final design looks similar to the InGen (Badshah et al., 2011) when folded. The shortest diameter of the knob status (hexagon) is around 71.6 mm. The longest diameter of the knob status is 78.6 mm. The length of the slider status is 181 mm. The width of the slider status is 60.5 mm including the sensor axis, and 30.2 mm excluding the axis. The height of device is 73.4 mm.

Figure 7 shows the pieces of KnobSlider. The device consists of forty pieces of the 3D printed case (yellow), five pulleys, a timing belt, a rotational sensor, five servo motors (blue, SG90). Figure 8 shows how motors and pieces are connected to create a hinge. Each motor opens and closes each hinge of the device by 60°. When all motors open and close, the device makes the straight shape for the slider state (Figure 5A) and round shape for the knob status (Figure 5C). Each motor is individually controlled by an Arduino (Figure 9). For sensing the knob or the slider value, we place a manufactured clickable knob^4^ at the base of the sensor center (Figures 6, 7). The sensor is connected to the bottom and top central gear, and used for both knob and slider states of the KnobSlider. When the hinges are closed, the block edges interlock with the bottom central gear making a knob state. User's rotation of the knob then translates to the sensor axis, and the device works as a knob. When the joints are open, the device makes a slider state. The central block is supported on the bottom central gear, but the rotation of the block does not affect the gear. Instead, the movement of the slider cursor is conveyed to the top central gear through the timing belt.

Table 3 summarizes how our prototype supports the requirements. The device can support two continuous parameters, and several can be used in parallel. The peak motor speed is 60° per 0.1 s, enabling the shape change in ~0.1 s. The KnobSlider can reach similar precision than knob/slider on their own; in our prototype, knob has 100 control positions per rotation, and slider has ~237 control positions per 112 mm (cursor traveling length). The knob diameter is ~75 mm. The outer length of the slider is around 182 mm, and the cursor's traveling distance is 112 mm due to the gears at the corners of slider. When it is a slider, the footprint is about 68.2 cm^2^. When it is a knob, the footprint becomes around 43.6 cm^2^. Additionally, the silicon base ensures stability. The KnobSlider has physical cursors for both knob and slider, but the slider friction varies because of the gaps between the blocks. Future engineering effort includes miniaturization, haptic feedback, cursor automation, and cable removal to ensure better mobility and multiturn knob. Even though the prototype is low fidelity, it is suitable to collect early feedback from users.

**Table 3:** Summary of how KnobSlider fulfills the design requirements.

**Requirements**	**Assessment**
R1. Many parameters	One KnobSlider accommodates two **continuous parameters in sequence**. Several KnobSliders can be used simultaneously.
R2. Fast interaction	Knob and slider interactions are as fast as **standard** ones. The shape change takes **0.1*****s***.
R3. Precise interaction	Knob: **100 control positions**/rotation, diameter is around **75 mm**. Slider: ~237 control positions/112 mm (cursor travel length). Silicon base sticks on the surface.
R4. Eyes-free interaction	KnobSlider offers **physical** knob, button and slider.
R5. Mobile interaction	KnobSlider is **small** enough so that several are available on a mobile surface.
R6. Retro-compatibility	KnobSlider provides **a button, knob and slider**.

*The text in bold highlights what features KnobSlider support the requirements*.

## Controlled Experiment: Pairwise Comparison

In a previous work we have studied the KnobSlider in a qualitative manner (Kim et al., 2018) and found that the participants had diverged opinion on the change of the shape. Some people did not mind the change of the shape, and some were surprised and felt they could get hurt by the device. Following this work our goal is now to investigate these effects through a controlled experiment. We are particularly interested in exploring user preferences on different speed conditions, and the preference difference according to the tangibility of the device—if the device is in the real world and tangible or it is on a video. We conduct one controlled study where we compared two conditions: in the first we used KnobSlider to show different speed properties to the participants; in the second condition, the participants only saw videos of shape-changing KnobSlider on a screen, with the same speed properties from the first condition.

### Rationale of the Experimental Design

The goal of our study is subjective in nature, and we choose to conduct a paired comparison experiment (David, 1963). It is a typical method used to gather Quality of Experience (QoE) feedback (Chen et al., 2009; Al Maimani and Roudaut, 2017; Serrano et al., 2017). Estimating preferences of objects based on subjective judgments is a critical step in psychological experiments with applications in many research fields such as marketing, environmental sciences, and health economics. It is widely used study to gather Quality of Experience feedback such as survey on product design preference, and also in many research fields where measure subjective judgement such as preferences and importance, including policy design, voting systems, and marketing (Jensen, 1986; Albers-Miller, 1996; Winner, 1999). The study is conducted by asking participants to choose between two conditions, mostly to choose the most preferable condition out of two. The experiment is designed to show all possible combinations of two conditions to the participants. Performing pairwise comparison ratings has been proven to produce more realistic results than asking for individual rankings (e.g., using a Likert scale; Bradley and Terry, 1952).

### Research Questions

In this study, we try to answer to the following questions.

**Q1. Is the preference with the physical device equivalent to the videos on a screen?** In this study, the observation of the shape-changes is limited to visual feedback and does not include interaction with the prototype, or other types of feedback. Hence, we believe that the preference of the physical prototype is transferable to screen medium. However, there can be an offset between the most preferred speed between the physical and the screen medium. For instance, users may prefer a little faster speed on the screen, because they know that the device cannot harm them.

**Q2. Do users prefer certain range of speed?** We expect that users would prefer a certain range of speed, not randomly any sort of speed. The preference will have a pattern such as a normal distribution.

**Q3. Which speed profile users prefer?** With the same maximum or average speed, people may prefer gradual speed change. We expect that users would prefer a speed profile that gradual changes the speed than one that instantly change the speed. I.e., they would prefer the device to gradually increase its speed from 0 to a certain amount, then gradually decrease the speed to 0, than the device to instantly change its speed from 0°/s to a certain amount, then stop. We assume so because gradual speed profile gives time to users to prepare themselves for the shape-changes.

### Experimental Design

**Figure 11:**
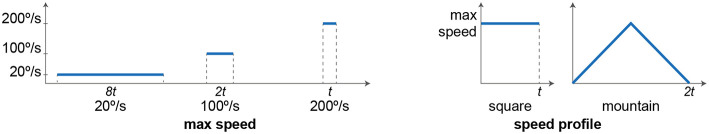
Two speed variables explored in the experiment. *Max speed* is the maximum speed that the motors will have over shape-changes. We use three amounts of *maximum speed, 20, 200*, and *100*°*/s*. *Speed profile* is the changes in the speed during shape-changes. *Square*: the speed of shape-change is constant over time. *Mountain*: the speed increases a constant acceleration until it reaches the maximum speed and decreases with the same absolute amount of the acceleration.

The study had three independent variables: *tangibility, max speed*, and *speed profile* (see Table 4). *Tangibility* refers to the fact that the shape-changes occur on the physical KnobSlider device or on a video. We choose to have *tangibility*, because studies of shape-changing interfaces choose to use either physical devices or videos (Strohmeier et al., 2016; Tiab and Hornbæk, 2016) and we do not know if they are equivalent and mutually substitutable. *Max speed* variable correspond to the maximum speed the shape-change of KnobSlider. We want to figure out between max aped and speed profile, which variable has more impact on preferences. We use three conditions—*20, 100, 200*°*/s* (Figure 11-left). They were distinguishable by users in a pilot study. The third variable *speed profile* was the dynamic of the shape-change: *square* and *mountain* (Figure 11-right). With the *square* profile, the motors changed their speed from 0°/s, to a maximum speed, and 0°/s over the shape-change. With the *mountain* profile, the motors linearly accelerated until reaching the maximum speed and then linearly decelerated until reaching 0°/s, resulting the speed making the mountain-like shape on the graph. This variable was to know if there is distinguishable preference on speed profiles when the maximum speed is the same.

**Table 4:** The variable and conditions of the controlled study.

**Variables**	**Conditions**	**Experiment design**
*Tangibility*	Device (tangible), video (non-tangible)	Between-subjects design
*Max speed*	20, 100, 200°/s	Within-subjects design
*Speed profile*	Square, mountain	Within-subjects design

*For the tangibility variable, we use between-subjects design to remove the learning effect. For the max speed and speed profile variables, we use within-subjects design to eliminate individual differences by participants on the variables*.

*Tangibility* was a between-subject variable, i.e., participants were randomly assigned to either the condition with physical KnobSlider or video but did not do both. We choose this variable to be between-subject to eliminate potential learning effect. The other variables were within-subject variables. With the Max Speed and the Speed profile, there were 6 permutation of 2 (_6_P_2_) so a total of 30 pairs to compare. The pairs were displayed in a randomized way using Latin Square design.

### Participants

We recruited 18 participants among the PhD students and researchers in the university (ages 14–59, avg. 29, SD 9.4, 5 females). We separated them into two groups for *tangibility* variable: nine people watched the physical device, and the rest nine people watched the videos on a screen. We surveyed the participants about the amount of experience with KnobSlider, technology adoption, and proficiency in using knobs and sliders. None of the participants have interacted with the device, and only three people have seen KnobSlider moving on video or for real. Around the half of the participants (11/18) answered that they are early majority in the technology adoption life cycle (Bohlen and Beal, 1957), the rest people answered as an innovator (1/18), early adopters (4/18), and late majority (2/18). The majority of the participants (14/18) had used knobs and sliders for simple interaction such as setting temperature with an oven dial. The rest of the participants responded having advanced experiences such as controlling sound parameters on a mixing board.

The participation for both studies was voluntary, and no compensation was offered. We followed a standard user study procedure in HCI where instructors show different types of interfaces and ask feedback from participants (e.g., Kwak et al., 2014; Tiab and Hornbæk, 2016). We explained the experiment procedure to the participants beforehand and acquired consent forms. They could withdraw their participation and their collected data at any point of the experiment or after it. There was no possibility of harming the participants, and the data was anonymized so that the person analyzing the data could not identify the participants. We gained ethics approval from our university ethics committee.

Note that for the first study (Kim et al., 2018), we picked professional users because we wanted to understand the current interaction, usages, and applications. This is a common practice used in the user-centered design process, which is a typical modus operandi from designers and HCI researchers. But for the controlled pairwise comparison study, our goal was to investigate perception of the movement and this could be studied with non-professional users, as both professional and non-professional users have not experienced shape-changing controls before and perception of the shape-changes should be the same for them. Having non-professional users also allowed us to have a broader range of participants and to increase the sample size for further statistical tests.

### Tasks

After an introduction of the experiment and obtaining informed consent, participants filled a short demographic questionnaire and started seeing the pairs of two conditions. They could take a break anytime between seeing pairs.

The participants were asked to sit in front of a table on which there was either the KnobSlider *device* (~30 cm distance from the end of the table) or *videos* on a screen (Figure 10). They could freely position themselves before the experiment. They were asked to fix their eyes on it and not to move on the chair once the experiments started. The physical device or the device on a video changed its shape from a slider to knob and back to slider. After watching each pair of shape-changes with two different speed properties (*max speed* and *speed profile*), the participants answered their preferences on a separate computer. The size of the device on the screen was similar to the actual device size (~12 cm), so that the size would not affect user preferences.

After seeing all the pairs, the instructor demonstrated how to use KnobSlider with an example application (light control on a stage). The participants then answered to qualitative questionnaire about the study. Each session lasted around 20 min.

### Results

We analyzed our results based on a literature that suggested a three-step analysis for pair-comparison studies (Chen et al., 2009). We show the analysis in the following sections.

#### Initial Responses

Among the 18 participants, five participants reported that they could not see a difference between some pairs, and one participant reported that s/he does not have a preference over a pair. This happened for nine pairs among 540 pairs (30 pairs × 18 participants), which was 1.6% of the responses. We assume that for some participants it was hard to see too fast movement. It occurred when the *max speed* of both conditions was equally high (*200*°*/s*) or when the average speed of both conditions was equally medium (*100*°*/s*), i.e., condition 1: *100*°*/s max speed* and rectangle *speed profile* (avg. speed of *100*°*/s*), condition 2: *200*°*/s max speed* and *mountain speed profile* (avg. speed of *100*°*/s*). We changed the responses to random as those participants would respond randomly when we force them to choose one of the conditions. We used discrete uniform distribution for the altered responses.

#### Individual Consistency

As the first analysis, we evaluated each participant's consistency on the responses by using Transitivity Satisfaction Rate (TSR). It quantifies how much the participants' preferences were transferred when answering different pairs. For example, a participant responded that speed condition A is more preferable than condition B and condition B is more preferable than condition C. If participant responds that s/he prefers A over C, we can say that the responses to the condition A, B, C are consistent. We used a program used in a previous literature (Al Maimani and Roudaut, 2017) to measure the individual consistency.

In the *device* condition, five participants showed the TSR of 1, which is the perfect consistency. Three participants had TSR between 0.71 and 0.75, meaning some disagreement. One participant showed TSR of 0.33. In the *video* condition, five participants had TSR above 0.8 (range from 0.82 to 1.0, avg. 0.92). The rest four participants' TSR ranged from 0.33 to 0.67. Our hypothesis about the inconsistency is that participants get used to faster speed of shape-changes more easily in the *video* condition, and they change their preferences.

#### Overall Consistency

We then evaluated the overall consistency of responses among the participants in each *tangibility* condition. We used Kendall's tau coefficient to calculate the overall consistency. We computed the ranking of preferred speed conditions for each participant, and then used calculated Kendall's tau coefficient, which shows how similar the rankings are. The participants in the *device* condition had Kendall's tau coefficient of 62%, and the participants in the *video* condition had Kendall's tau coefficient of 75.3%. It shows that there is positive correlation in the participants' preferences, not negative correlation, i.e., the participants liked the speed properties in a similar order, not an opposite order.

#### Answers to the Research Questions

**Figure 12:**
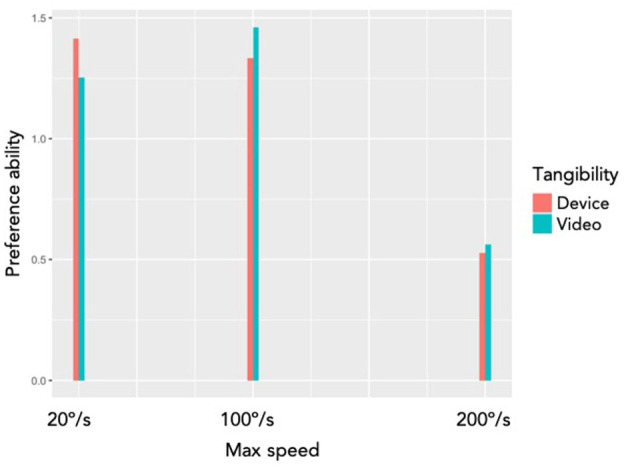
The Bradley-Terry-Luce model output of different *max speed* and *tangibility* variables. The red bars show the preference ability of *physical device* (*tangible*) condition with different conditions of *max speed*, and the green bars show the preference ability of *video* (*non-tangible*) condition of the same device with different conditions of *max speed*.

**Figure 13:**
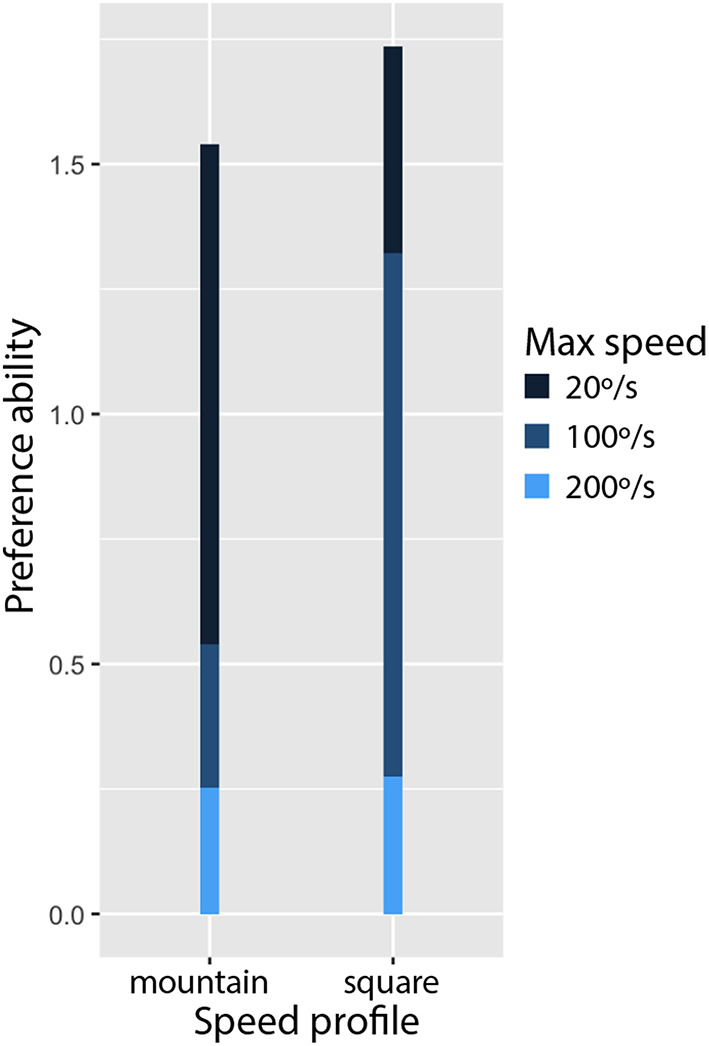
The Bradley-Terry-Luce model output of *speed profile* variable. The participants preferred the *square* profile over the *mountain* profile, when the *max speed* was accumulated.

In this section, we answer to the research questions by analyzing the results. We used Bradley-Terry-Luce model (Bradley and Terry, 1952) to compute the “ability” of the conditions that have been compared in the study. Figure 12 shows the ability of *max speed* conditions and Figure 13 shows the ability of *speed profile* conditions.

**Q1. Is the preference with the physical device equivalent to the videos on a screen?** The participants preferred different max speed depending on the *tangibility* variable (Figure 12). With the *device*, the participants most preferred the lowest speed (20°/s), and with the *videos*, the participants most preferred the medium speed (100°/s). It means that using videos for perceptional study on shape-changing interfaces (Pedersen et al., 2014; Tiab and Hornbæk, 2016) can result different conclusion than using physical devices.

**Q2. Do users prefer certain range of speed?** Users preferred a certain range of speed, instead of preferring all range of speed. The result is illustrated in Figure 12. We observed very the least preference on the highest speed (*200*°*/s*). It reassures the result from our qualitative study (Kim et al., 2018) that some users got surprised by the shape-changes of KnobSlider. Users do not like surprising feelings caused by the fast movements of the device.

**Q3. Which speed profile users prefer?** Figure 13 shows the users preferences on the *speed profile* conditions, *square* and *mountain*. Surprisingly, they preferred the *mountain* profile over the *square* profile, regardless of the *max speed*. It was not consistent with the fact that the *mountain* profile reduced the average speed of shape-changes with the highest *max speed* (*200*°*/s*) to the half (*100*°*/s*), and that the participants preferred the medium *max speed* (*100*°*/s*) than the highest *max speed* (*200*°*/s*). It might be related to that some participants mentioned too slow shape-changes were “boring.” They explained that they could know the device would change its shape once it started moving. In the same sense, we can hypothesize that the first acceleration part of the *mountain* profile made the participants prepared for shape-changes with any *maximum speed*, and they felt bored over shape-changes with the *mountain* profile. It would be interesting to investigate if it is more acceptable to have gradual speed change only at the beginning of shape-changes.

## Discussion and Future Work

Although we chose the design of KnobSlider from the evaluation of low-fidelity prototypes, the folding design raised ergonomic issues on the device. For example, users cannot rest their hands on the device during shape-changes, because the folding and unfolding of the device can hit their hands. A future study should consider ergonomic aspects of shape-changes such as where users' hand should rest during shape-changes, in addition to the design requirements from the formative study. Such studies have not been done because shape-changing interfaces are relatively new, and most studies have been focusing on fabrication methods of the interfaces. It would be interesting to explore dynamic ergonomics, how the kinetic parameters of shape-changing interfaces—e.g., speed, path, direction, and space (Rasmussen et al., 2012)—would affect ergonomics. From the observations in our study, we suggest future designs of shape-changing interfaces not to intervene the grasp of the device through shape-changes.

Due the technical limitation, the implementation of KnobSlider became larger than we expected and the height of the knob status forced the users not to rest their arms or palms when rotating it. Also, the width of the knob status forced them to grasp it from the top and rotate with their entire hands, while in the formative study they mostly approached the small dials from the side and used two fingers only. A future study should make the device to have similar dimension to existing knobs and sliders, so that users can keep their micro-gestures on the devices. Lastly, the hinges of the device can affect ergonomics. It could be uncomfortable for users if they put their fingers at the hinges while the hinges are closing.

Tangibility affects the preference on speed. Users preferred slower shape-changes with the physical device and faster shape-changes with videos. It means that studies using videos for perceptional studies should consider the difference from the *tangibility* variable. To enable researchers to freely use videos instead of physical devices when they have limited resources, future studies can find the mathematical model of speed preferences on tangibility. For example, the participants in our study preferred the shape-changes on videos four times faster than the shape-changes on the device. Future work can further verify how much faster speed is preferred on videos than physical devices.

We also learned that users do not like fast shape-changes on a shape-changing device. In the controlled experiment, we explained that the device is to control parameters and hence users need to touch it when they use it. It could have made the participants to consider negatively fast shape-changes. Some shape-changing interfaces used shape-changes for display and did not require input from users (Ishii et al., 2001; Thomsen, 2009). It would be interesting to investigate whether the necessity of touching shape-changing interfaces would affect the speed preferences or not. If the device was for display, the result might have been different. A future study can investigate the effect of users' interactivity and the user-device distance on speed preferences.

Lastly, the users showed that they are prepared themselves once they observe the beginning of the shape-changes. We can further explore different modalities to inform users before shape-changes such as sound, visual display, or direct shape-change, and their design space such as speed and intensity. It would be interesting if users' preferences on the design space stay consistent regardless of the modalities.

## Conclusion

In this paper, we conducted a formative study to understand professional users' need on parameter control interfaces. We derived five design requirements and used them to evaluate our nine low-fidelity prototypes. We then implemented KnobSlider, a shape-changing device can be a knob or slider, based on the best low-fidelity prototype. The controlled study of speed preference with KnobSlider shows that speed preference is not the same between physical device and videos on a screen. It suggests that future studies using videos of shape-changing interfaces should consider this difference when they plan experiments.

## Data Availability

The datasets generated for this study are available on request to the corresponding author.

## Ethics Statement

The participation to our user studies were voluntary, and the procedures of the studies and the types of data collected were informed to the participants before the studies. We respected the participants' confidentiality and anonymity during the recording and processing of the data. We gained ethics approval from the University of Bristol for the second user study (Id nr.: 211672).

## Author Contributions

HK, CC, and AR contributed to the conception of the article. CC contributed the formative study. HK, CC, and AR contributed to its analysis. HK contributed to the device implementation. HK, CC, and AR contributed the planning and conception of the controlled experiment. HK contributed to the execution of the experiment and analysis of the data.

### Conflict of Interest Statement

The authors declare that the research was conducted in the absence of any commercial or financial relationships that could be construed as a potential conflict of interest.

## Footnotes

^1^https://www.bls.gov

^2^e.g. https://www.thingiverse.com

^3^https://www.thingiverse.com/thing:1276095

^4^griffintechnology.com/us/powermate
